# Editorial: New challenges and future perspectives in pathological conditions

**DOI:** 10.3389/fnbeh.2023.1201044

**Published:** 2023-05-26

**Authors:** Mustapha Muzaimi, K. N. Bhanu Prakash, Pike See Cheah, Linqing Feng

**Affiliations:** ^1^Department of Neurosciences, School of Medical Sciences, Universiti Sains Malaysia, Kubang Kerian, Malaysia; ^2^Cellular Image Informatics Division, Bioinformatics Institute (A^*^STAR), Singapore, Singapore; ^3^Faculty of Medicine and Health Sciences, Universiti Putra Malaysia, Serdang, Malaysia; ^4^Research Center for Augmented Intelligence, Zhejiang Lab, Hangzhou, China; ^5^College of Biomedical Engineering and Instrument Science, Zhejiang University, Hangzhou, China

**Keywords:** alcohol use disorder, schizophrenia, Angelman syndrome, palmitoylethanolamide, acupuncture, CIBP, Huntington's disease, N-acetylcysteine

Over the last decade, there have been significant findings and advances, from a preclinical or translational perspective, on the impact of inherent or acquired disorders, diseases, or conditions affecting behavior, with an emphasis on the underlying mechanisms of fundamental aspects of health and disease. In this Research Topic, we feature eight articles (four original research papers, three brief/case reports, and one narrative review) with contributions from 52 authors.

Substance use disorder remains a challenging global health burden. Among the numerous substances of abuse, alcohol use disorder (AUD) is among the most prevalent. This is partly due to an incomplete understanding of the molecular mechanism that underlies AUD, which limits the therapeutic approaches as well as the emerging epigenetic evidence for the compulsive tendency in susceptible individuals, albeit other confounders such as sociocultural and traumatic life events are well-understood (Nestler and Lüscher, 2019). In this context, Jarczak et al. critically summarized the potential roles of epigenetic modifications (DNA methylation and repair) over a lifetime that may influence individuals' AUD susceptibility. The current progress in DNA methylation biology and technique could catalyze the role of biomarkers for AUD diagnosis, prognosis, and prospects for epigenetic drug therapy. Jarczak and team highlighted the need for robust animal models for this disorder, refining the causative role of differentially methylated genes in human addiction pathophysiology and their relationship to other AUD-related behaviors.

The adverse effects on behavior due to motor and/or cognitive impairments that disrupt movements and cognition are well-recognized in a wide range of neurological conditions (Bloch-Gallego and Anderson, 2023; Gupta et al., 2023). Such impairments are often attributable to conditions such as pain-related pathological conditions, or as a part of neurodevelopmental disorders. Ji et al. and Negrón-Moreno et al. reported on methods to expand specific design on motor impairment seen in the cancer-induced bone pain (CIBP) condition and Angelman syndrome (AS), respectively. For CIBP, Ji et al. presented a model of metastatic CIBP caused by an allograft of Lewis lung cancer cells in male C57BL/6 mice aged 8 weeks involving the femoral medulla cavity to record the pain and motor behavior. The CIBP mice suffered severely impaired motor coordination as shown by open-field and rotarod tests. Of note, the latency on rotarod declined in CIBP mice while the distance in open filed test remained unchanged, which suggests a dominant effect on motor coordination that raises further research opportunities on the underlying pathomechanism for pain and locomotion, such as a peptidergic pathway. Overall, the brief report provided new insights for research on the mechanisms responsible for impaired motor behavior in the setting of CIBP. Angelman syndrome (AS) is a monogenic neurodevelopmental disorder caused by the UBE3A gene mutation, which results in symptoms such as seizure, speech disturbance, and motor and cognitive impairment. Negrón-Moreno et al. provided a detailed protocol to assess cognitive behaviors in a well-known AS mouse mutant model for motor impairments, Ube3a^m−/p+^, using 5-choice serial reaction task time (5CSRTT). The Ube3a^m−/p+^ mice appeared to have more omissions during 5CSRTT not only during training compared to littermate controls, but also showed motor impairments in the open field and delayed access to pellet reward. Interestingly, the mice had normal latency in reward retrieval despite hypoactivity during habituation, and an increase in omissions is unlikely due to the concurrent impaired motor functions. Hence, this work highlighted the likelihood of behavioral aspects being influenced by motor impairments and the usability of the 5CSRTT protocol in this perspective for future AS research, particularly in evaluating attention and impulsivity and complementing existing behavioral modalities for AS Ube3a mice model.

The use of rodent models as preclinical models of human neuropsychiatric and development disorders remains essential as part of the therapeutic strategy. Bühner et al. utilized a cyclin-D2 knockout mouse model (CD2-KO) that mimicked schizophrenia prodrome to test the therapeutic potential of repurposable chronic N-acetylcysteine (Skvarc et al., 2017) treatment in drinking water during development for non-psychotic symptom related to cognitive and affective deficits in the prodromal phase of schizophrenia. Cognitive function deficit may also accompany mild traumatic brain injury (mTBI) as reported by Bruhns et al. in a mouse that was ameliorated by the use of angiotensin-(1-7) to reduce brain inflammation in this condition. From a closed skull, single injury model for mTBI, male mice were treated with Ang-(1-7) vs. saline control for 5 days post-injury before being tested on their cognitive and motor functions via novel object recognition. The tests were conducted initially daily for 5 days and later on day 18. Ang-(1-7) daily for 5 days post-mTBI significantly increased cognition with less structural damage found to the cortical and hippocampus of the treated mice, hence offering a novel therapeutic prospect in mitigating long-term cognitive impact following mTBI.

In another setting, the use of acupuncture to treat post-ischaemic stroke sequelae offers an opportunity to bridge the translational gap in stroke. Yang et al. highlighted the therapeutic potential of traditional and electroacupuncture (EA) on rat models, comparing five animal groups with the sham operation, model, traditional acupuncture, EA, and drug (edaravone) therapies. Following measurements from days 1, 3, 5, and 7 after reperfusion, both acupuncture and EA (at GV20 and ST36) significantly minimized neurological deficits, as shown by improved functional scores, comparable to that of edaravone, although the underlying mechanism warrants further research. Herrera et al. tested palmitoylethanolamide (PEA), a compound previously shown to be neuroprotective in experimental brain injury and neurodegeneration models (Scuderi et al., 2014; Beggiato et al., 2019). They tested on newborn rat models with induced perinatal asphyxia (PA). This condition results in marked neurodevelopmental delay, i.e., an immature brain, and the research aimed to show the early protective role of PEA in the experimental PA rat model. PEA treatment resulted in the reduction of PEA-induced hippocampal damage and restoration of selected neurobehavioral responses such as gait, placing, and grasp reflexes in the treated group.

Phillips et al. highlighted a case report of a 41-year-old patient with a familial and degenerative condition, Huntington's disease (HD), in which metabolic therapy that targets mitochondrial dysfunction (Singh et al., 2021) was being tested. The patient underwent a time-restricted ketogenic diet (TRKD) for 48 weeks that resulted in stark improvements in his motor symptoms, HD rating scale, activities of daily living, and HD-related behavioral symptoms although cognitive function remained unchanged. TKRD did not result in any weight change and no significant adverse effects were reported throughout the therapy period. This HD-TRKD therapy combination is the first of its kind and it is understood that the patient remains steadfast as it proves beneficial for his condition.

In summary, the current Research Topic includes original studies, a narrative review, and case reports regarding novel molecular mechanisms, preclinical models to improve our understanding of pathophysiology, and potential therapeutic candidates for several neurological, neuropsychiatric, and neurodegenerative disorders. We extend our sincere gratitude to the Editorial Office of Frontiers in Behavioral Neuroscience, the authors, and the reviewers for their invaluable contributions to this Research Topic.

## Author contributions

MM drafted the manuscript. KP, PC, and LF collaborated on the topics. All authors contributed to the article and approved the submitted version.
